# Complete mitochondrial genome of yellowback sea bream *Evynnis tumifrons*

**DOI:** 10.1080/23802359.2016.1144095

**Published:** 2016-06-20

**Authors:** Lin Zeng, Lihua Jiang, Meiying Xu, Changwen Wu

**Affiliations:** National Engineering Research Center of Marine Facilities Aquaculture, Zhejiang Ocean University, Zhoushan, Zhejiang, China

**Keywords:** *Evynnis tumifrons*, mitochondrial genome, phylogenetic tree

## Abstract

The complete mitochondrial genome of the *Evynnis tumifrons* was 16 616 bp in length and contained 13 protein-coding genes, 22 transfer RNA genes and 2 ribosomal RNA genes. The overall base composition of *E. tumifrons* was A 27.05%, T 26.42%, C 29.45% and G 17.07%. Phylogenetic tree construction indicated that *Evynnis tumifrons* was most closely related to *Parargyrops edita* and *Pagrus auriga*. This molecular information will contribute to better understand its evolution and population genetics.

*Evynnis tumifrons*, which belongs to Perciformes, Sparidae, is one of the most economically marine fish (Tominaga et al. 2005). *Evynnis tumifrons* has previously been synonymized with *Taius tumifrons*. It is a fairly widespread species in the northwest Pacific (Iwatsuki et al. 2007). In order to obtain genetic information and understand the evolution of *E. tumifrons*, we sequenced the mitochondrial genome of *E. tumifrons* (GenBank accession no. KT724963).

Evynnis *tumifrons* were obtained from the otter trawl in east town, Zhoushan city, Zhejiang province (30°19′52.40″N, 122°71′30.09″E). Initially, the fish were identified based on both the morphological features and the *COX1* mitochondrial gene. Tissue samples for molecular analysis were reserved in absolute ethyl alcohol. The complete mitochondrial genome of *E. tumifrons* was extracted from muscle tissue using the phenol–chloroform method. The PCR products were sequenced by Sanger’s method.

The complete mitochondrial genome of *E. tumifrons* was 16 616 bp in length, containing 13 protein-coding genes, 22 transfer RNA genes (tRNA) and two ribosomal RNA genes (rRNA) (Table 1). The mitogenome base composition was A 27.05%, T 26.42%, C 29.45% and G 17.08%, A + T content (53.47%) was slightly higher than the G + C (46.53%) content. Thirteen protein-coding genes can be classified into two categories: *ND1, ND2, COX1, COX2, ATP8, ATP6, COX3, ND3, ND4L, ND4, ND5* and *CYTB* were encoded by the light strand, only *ND6* was encoded by the heavy strand. Ten protein-coding genes (*ND1, ND2, COX2, ATP8, ATP6, COX3, ND3, ND4L, ND5* and *CYTB*) started with an ATG initiation codon, *COX1* and *ND4* started with a GTG initiation codon, while *ND6* used CAT as the initiation codon. Six protein-coding genes (*ND1, ND2, ATP8, ATP6, ND4L* and *ND5*) used TAA as the termination codon, *COX3* used TAG as the termination codon, *ND6* used TTA as the termination codon, while five genes (*COX1, COX2, ND3, ND4* and *Cytb*) showed an incomplete stop codon, which were similar to other fishes (Xia et al. 2007; Ma et al. 2015). The two ribosomal RNA genes, 12SrRNA (955 bp) was located between *tRNA^Phe^* and *tRNA^Val^* genes, and 16SrRNA (1699 bp) was located between *tRNA^Val^* and *tRNA^Leu^* genes.

**Table 1.** Mitochondrial genome characteristics of *Evynnis tumifrons.*

Gene	Position		Size(bp)		Codon				
	From	To		Nucleotide	Amino acid	Initiation	Stop	Intergenic nucleotides^a^	Strand^a^
tRNA-Phe	1	68		68					
12S rRNA	69		1023	955					
tRNA-Val	1024		1095	72					
16S rRNA	1096		2794	1699					
tRNA-Leu	2795		2867	73					
nad1	2868		3842	975		ATG	TAA		L
tRNA-Ile	3848		3917	70				5	
tRNA-Gln	3916		3987	72				−2	
tRNA-Met	3987		4056	70				−1	
nad2	4057		5103	1047		ATG	TAA		L
tRNA-Trp	5103		5172	70				−1	
tRNA-Ala	5173		5241	69					
tRNA-Asn	5243		5315	73				1	
tRNA-Cys	5351		5416	66				35	
tRNA-Tyr	5417		5486	70					
COX1	5488		7036	1549		GTG	T– –	1	L
tRNA-Ser	7041		7111	71				4	
tRNA-Asp	7114		7186	73				2	
COX2	7195		7885	691		ATG	T– –	8	L
tRNA-Lys	7886		7960	75					
ATP8	7962		8129	168		ATG	TAA	1	L
ATP6	8120		8803	684		ATG	TAA	−10	L
COX3	8803		9588	786		ATG	TAG	−1	L
tRNA-Gly	9588		9659	72				−1	
nad3	9660		10 008	349		ATG	T– –		L
tRNA-Arg	10 009		10 078	70					
nad4L	10 079		10 375	297		ATG	TAA		L
nad4	10 369		11 749	1381		GTG	T– –	−7	L
tRNA-His	11 750		11 818	69					
tRNA-Ser	11 819		11 887	69					
tRNA-Leu	11 894		11 966	73				6	
nad5	11 967		13 805	1839		ATG	TAA		L
nad6	13 802		14 323	522		CAT	TTA	−4	H
tRNA-Glu	14 324		14 392	69					
cytb	14 397		15 537	1141		ATG	T– –	4	L
tRNA-Thr	15 538		15 610	73					
tRNA-Pro	15 610		15 679	70				−1	

a Negative numbers indicate overlapping nucleotides. Numbers correspond to the nucleotides separating adjacent genes. L indicates the light strand and H indicates the heavy strand.

In the mitochondrial phylogeny, *E. tumifrons* was most closely related to *Parargyrops edita* and *Pagrus auriga*. *Pagrus major* was grouped with *E. tumifrons*, *Parargyrops edita* and *Pagrus auriga*; *Rhabdosargus sarba*, *Sparus aurata*, *Acanthopagrus schlegelii* and *Acanthopagrus latus* were clustered together; the remaining six species formed another large cluster (Figure 1).

**Figure 1.**
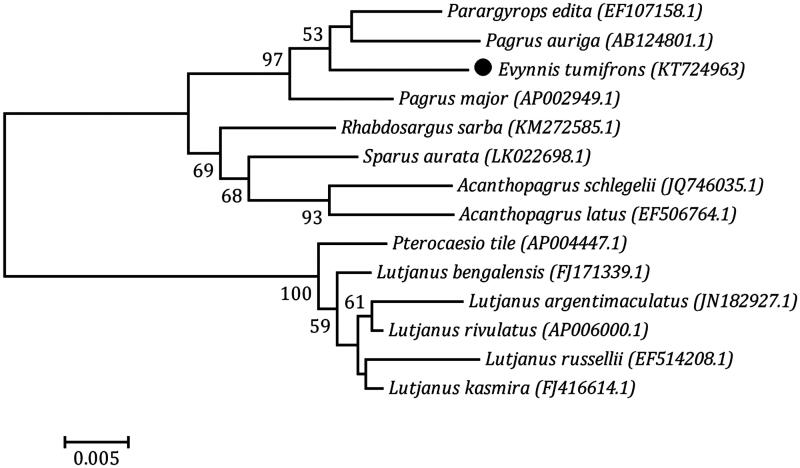
Phylogenetic tree of *Evynnis tumifrons* was constructed with the neighbor-joining method using the program MEGA 4.0 (MEGA Inc., Englewood, NJ). The numbers at each branch indicate the percentage bootstrap values
